# Novel cationic cryptides in *Penaeus vannamei* demonstrate antimicrobial and anti-cancer activities

**DOI:** 10.1038/s41598-023-41581-9

**Published:** 2023-09-06

**Authors:** Amr Adel Ahmed Abd El-Aal, Fairen Angelin Jayakumar, Chandrajit Lahiri, Kuan Onn Tan, Kavita Reginald

**Affiliations:** 1 Department of Biological Sciences, School of Medical and Life Sciences, Sunway University, 47500 Bandar Sunway, Selangor Malaysia; 2 Marine Microbiology Lab., National Institute of Oceanography and Fisheries (NIOF), Alexandria, 84511 Egypt; 3 Present Address: Department of Biotechnology, Atmiya University, Rajkot, Gujarat 360005 India

**Keywords:** Cancer, Computational biology and bioinformatics, Drug discovery, Microbiology

## Abstract

Cryptides are a subfamily of bioactive peptides that exist in all living organisms. They are latently encrypted in their parent sequences and exhibit a wide range of biological activities when decrypted via in vivo or in vitro proteases. Cationic cryptides tend to be drawn to the negatively charged membranes of microbial and cancer cells, causing cell death through various mechanisms. This makes them promising candidates for alternative antimicrobial and anti-cancer therapies, as their mechanism of action is independent of gene mutations. In the current study, we employed an in silico approach to identify novel cationic cryptides with potential antimicrobial and anti-cancer activities in atypical and systematic strategy by reanalysis of a publicly available RNA-seq dataset of Pacific white shrimp (*Penaus vannamei*) in response to bacterial infection. Out of 12 cryptides identified, five were selected based on their net charges and potential for cell penetration. Following chemical synthesis, the cryptides were assayed in vitro to test for their biological activities. All five cryptides demonstrated a wide range of selective activity against the tested microbial and cancer cells, their anti-biofilm activities against mature biofilms, and their ability to interact with Gram-positive and negative bacterial membranes. Our research provides a framework for a comprehensive analysis of transcriptomes in various organisms to uncover novel bioactive cationic cryptides. This represents a significant step forward in combating the crisis of multi-drug-resistant microbial and cancer cells, as these cryptides neither induce mutations nor are influenced by mutations in the cells they target.

## Introduction

Cryptides are short bioactive peptides embedded latently into a larger precursor or parent protein^1^. These cryptides are liberated from their parent proteins by proteolytic cleavage during the activation of cellular processes in the human body^2^. The decrypted sequences play crucial roles in many biological processes, including cardioprotective and immunomodulatory functions, and exhibit bioactivities against a wide range of pathogens and cancer cells. Moreover, cryptides could be liberated from their precursors in all eukaryotic cells as a defense mechanism against invasive infections by the proteolytic effects of host and/or pathogen enzymes^3^. Cryptides were classified into three classes; type 1 cryptides are naturally liberating from their precursors via proteolytic reactions and demonstrate unrelated bioactivities to those exhibited by their precursors, type 2 cryptides are similar to type 1 in terms of the naturally occurring but they possess relative bioactivities to their parent proteins and type 3 cryptides are in vitro decrypted regardless their functional relationship with their parent proteins^3^. Mining for peptides with low molecular weights is a well-known strategy to discover type 1 and 2 cryptides from their natural sources while the enzymatic digestion of biological samples is a common strategy for type 3 cryptides discovery. Microbial fermentation, digestive enzymes, and plant and microbial proteases are commonly used for this purpose^4^. Mass spectrometry-based cryptomic study is a powerful strategy that was used to identify cryptides from biological sources before testing their bioactivities in vitro. For example, Electrospray ionization-Fourier transform mass spectrometry (ESI-FTMS) was used along with ultra-high pressure liquid chromatography in a combination to which referred as (UHPLC) ESI-FTMS in the identification of 1100 cryptides from almost 200 parent proteins from the whole cell lysate of *Saccharomyces cerevisiae*, and in the identification of 200 cryptides from 29 parent proteins in the plasma^2^. Moreover, machine learning-based approaches were recently used to discover novel antimicrobial cryptides from the human proteome^5^.

Cationic antimicrobial peptides (CAMPs) generally possess selective broad-spectrum antimicrobial and anti-cancer activities due to their electrostatic interaction with the negatively charged bacterial and cancer cell membranes^6^. This mechanism causes functional or structural membrane alteration by disturbing the lipid bilayer, thereby affecting membrane permeability^7^. Moreover, they possess a non-membranolytic mechanism in which they penetrate the cell membrane and interact with the negatively charged intracellular molecules such as nucleic acid and phosphorylated proteins in bacterial cells^7,8^. Likewise, they can penetrate the cancer cell membranes, affect the mitochondrial membrane, and interact with the nuclear DNA, which subsequently induces apoptosis and could promote autophagy^9,10^. Due to the varied and selective mechanisms of action of the cationic peptides, they have been developed as a new potential therapeutic strategy with many completed or in progress clinical trials investigating their antimicrobial and antineoplastic effects (www.clinicaltrials.gov). For example, DPK-60 was tested against external otitis and atopic dermatitis bacterial infections (NCT01447017 and NCT01522391), LTX-109 was tested against *S. aureus* nasal infection, Gram-positive skin infections and impetigo (NCT01158235, NCT01223222, and NCT01803035) and hlF1-11 against bacterial and fungal infections (NCT00430469). Moreover, many completed and ongoing clinical trials study the anti-cancer effects of LTX-315 alone or in combination with other medications such as NCT01986426, NCT01058616, and NCT01223209. The dose-dependent intratumorally injection of LL37 in melanoma patients was investigated (NCT02225366). Thus, many studies have pointed out the potency of CAMPs, alone or in combination with other drugs, as a potential alternative to the current strategies that have contributed to the high rate of resistance and subsequent therapeutic failure^11,12^.

The ocean covers almost 70% of our planet and comprises 50–80% of the global biodiversity; hence it is a potential source of bioactive compounds, including AMPs^13,14^. Due to the marine ecosystem’s harsh nature, marine-derived AMPs may possess higher stability that renders them compatible with physiological salt, serum, and pH conditions^14^. For example, Pleurocidin is an AMP isolated from a marine flatfish *Pseudopleuronectes americanus* (Winter flounder) and has been reported to be active against bacterial pathogens at high NaCl concentrations, up to 625 mM^15^. This potential salt-insensitivity of marine-derived AMPs gives them an advantage over many other cationic AMPs, which may be totally or partially inactivated under the physiological salt concentrations, such as definiens, magainins, and indolicidins^16^.

Shrimps depend on the innate immune system that partly utilises AMPs to defend against microbial invasions^13^. Many gene-encoded AMP families with antimicrobial activities were identified in shrimps, for instance, Penaeidins, Crustins, Anti-lipopolysaccharide factors (ALF), and Stylicins^17^. Furthermore, several other antimicrobial peptides (AMPs) demonstrate anti-cancer properties. For instance, the peptide B11 derived from hemocyanin has been found to have an impact on the human cervical cancer cell line (HeLa), human hepatocellular carcinoma (HepG2), and human esophageal cancer cell line (EC109)^18^. Taken together, shrimps have the potential to be a valuable source of gene-encoded AMPs that could serve as models for the virtual discovery of new cryptides. These cryptides could represent a possible answer to the current threat posed by multi-drug-resistant microbial and cancer cells, given that their effectiveness is not influenced by these cells’ mutations^19^.

The traditional discovery of cryptides is based on trial-and-error techniques that are costly, time-consuming, and labour-intensive. In addition, the drug development process might be hampered due to the low activity or the high toxicity of the discovered peptides^20^. To overcome these challenges, we used in silico techniques to identify novel bioactive cryptides. We achieved this by re-mining an online RNA-seq dataset at the National Center for Biotechnology Information-Sequence Read Archive (NCBI-SRA) using open-source and user-friendly bioinformatics applications. The identified cyrptides were then synthesised and tested for their potential antimicrobial and anti-cancer activities.

## Results

### Computational identification and characterisation 

The functional annotation of the 28,445 longest isoforms obtained from the de novo transcriptome assembly of the SRP126153 dataset resulted in the identification of 6246 (22%) gene-encoded AMPs by BLASTx. Further filtration of the annotated hits by keywords resulted in 802 gene-encoded AMPs in shrimp that were considered potential precursors of bioactive cyrptides. Then, 12 (~ 1.5%) gene-encoded AMPs (precursors), which had not been published, were selected for further study. Trinity assembly statistics, nucleotide, and amino acid sequences, as well as the annotation of the selected precursors, are included in the supplementary data. We identified the highest-scored bioactive 15 amino acids encrypted segment in silico within each precursor as AD1-AD12 (Table 1). The potential antimicrobial and anti-cancer activities of these twelve cryptides were next predicted by several bioinformatics algorithms. Eight of the 12 peptides were predicted to have antimicrobial activity besides having non-hemolytic and non-toxic properties against healthy cells, except for AD1, AD2, AD4, and AD5. Nine cryptides were predicted to have anti-cancer activities (Table 1). As cryptides would need to penetrate the cell membrane to exert their activity, we further selected cryptides with a net positive charge ≥  + 3, resulting in a final selection of five cryptides, AD4, AD7, AD8, AD11, and AD12, for the in vitro characterisation assays.

**Table 1:** In silico identification and characterisation of cryptides.

Trinity ID	Cryptide ID	Sequence	MW (Da)	Net Charge	IEP	HP (%)	AMA	ACA	Toxicity	Hemolysis	CPA
DN1899	AD1	**KCC**FDRCFE**R**HV**C**KF	1921.3	+ 2.25	8.5	53	AMP	Non-ACP	Toxic	Non-hemolytic	Non-CPP
DN35987	AD2	**KCC**FDT**CL**NHHT**CKL**	1766.1	+ 1.50	7.9	46	AMP	ACP	Toxic	Non-hemolytic	Non-CPP
DN8354	AD3	**GR**WTA**G**SHS**G**T**G**A**G**S	1388.4	+ 1.25	9.7	20	AMP	ACP	Non-toxic	Non-hemolytic	Non-CPP
DN34505	AD4	**R**H**CLR**S**KR**PPNV**C**PH	1800.1	+ 4.50	10.8	26	AMP	ACP	Toxic	Non-hemolytic	CPP
DN1553	AD5	**CR**TPV**G**YV**CC**KP**GRC**	1642.0	+ 2.80	8.9	40	AMP	Non-ACP	Toxic	Non-hemolytic	Non-CPP
DN10332	AD6	**RG**ESNT**R**S**K**S**G**VVNA	1561.6	+ 2.00	10.8	20	AMP	ACP	Non-toxic	Non-hemolytic	Non-CPP
DN2676	AD7	F**LR**W**RLK**F**K**S**K**VW**C**P	1994.4	+ 5.00	11.1	53	AMP	ACP	Non-toxic	Non-hemolytic	CPP
DN13227	AD8	**G**HY**C**NFSVTP**K**F**KR**W	1870.1	+ 3.25	9.8	33	AMP	ACP	Non-toxic	Non-hemolytic	CPP
DN4554	AD9	VITAA**K**AA**K**DFVV**R**A	1559.8	+ 2.00	10.0	66	AMP	ACP	Non-toxic	Non-hemolytic	Non-CPP
DN19134	AD10	AI**K**DFV**K**QAVI**KG**IM	1661.0	+ 2.00	9.7	60	AMP	ACP	Non-toxic	Non-hemolytic	Non-CPP
DN468	AD11	**RL**Q**L**NY**KGK**MW**C**P**G**W	1880.2	+ 3.00	9.8	40	AMP	Non-ACP	Non-toxic	Non-hemolytic	CPP
DN63324	AD12	FFA**L**Q**C**AA**K**T**R**T**RR**V	1768.1	+ 4.00	11.7	53	AMP	ACP	Non-toxic	Non-hemolytic	CPP

Cryptides were identified from their precursors by AntiBP online tool. Then the identified cryptides were characterised by Expasy online tool and antimicrobial peptide database (APD3).

*MW* molecular weight, *IEP* isoelectric point, *HP* hydrophobicity, *AMA* antimicrobial activity, *AMP* antimicrobial peptide, *ACA* anticancer activity, *ACP* anticancer peptide, *CPA* cell penetration ability, *CPP* cell penetrative peptide.

**Figure 1:**
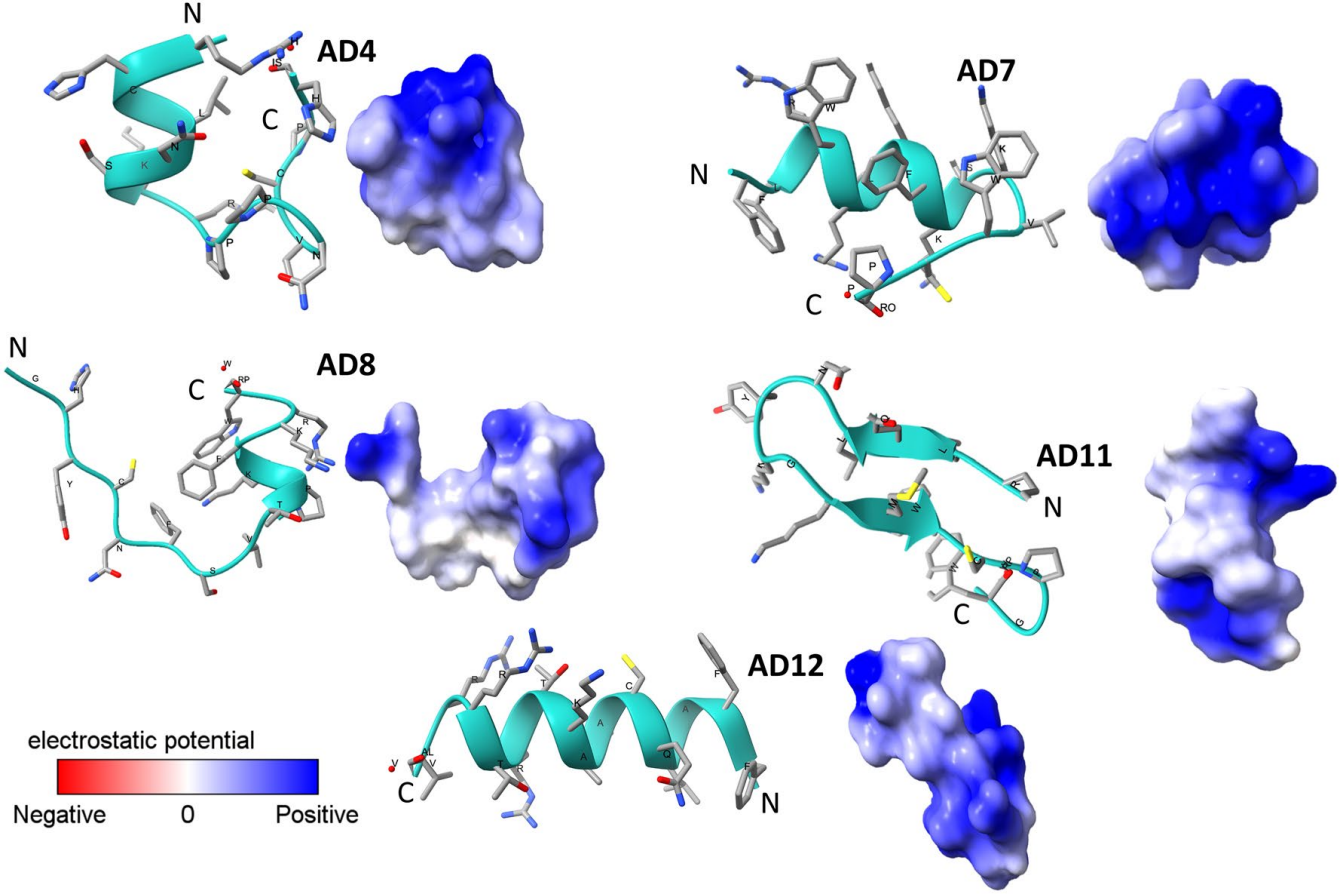
The predicted 3D structures of the identified cryptides. The selected cryptides were subjected to PepFold3 for de novo 3D structural prediction. The predicted structures and the electrostatic potential on their surfaces were visualised by ChimeraX software.

The predicted 3D structures of the chosen cryptides are illustrated in Fig. 1. AD4 showed almost two turns of right-handed α helical structure that compiles the first seven residues at the N-terminal. In comparison, the last eight residues at the C-terminal showed an extended structure. Likewise, AD7 revealed almost 2.5 turns of right-handed α helical structure from the second amino acid residue (Leucine) to the tenth one (Serine) at the C-terminal. However, the first and the last five residues were predicted to form an extended structure at both N-terminal and C-terminal, respectively. In contradiction to AD4 and AD7, the first eight residues at the N-terminal as well as the last three residues at the C-terminal of AD8, are predicted to form extended shapes while from the ninth (Threonine) to the twelfth (Phenylalanine) residue were predicted to form almost one right-handed α helical turn. Moreover, AD11 is predicted to form a typical two antiparallel β-sheet strands and AD12 as a right-handed α helical structure that compiles all residues except the last two at the C-terminal (Arginine and Valine), which are shown as a short extended structure. The molecular electrostatic potential (MEP) was represented with a colour scale indicating negative and positive extremes (Fig. 1).

### Antimicrobial activity of the selected cryptides

**Figure 2:**
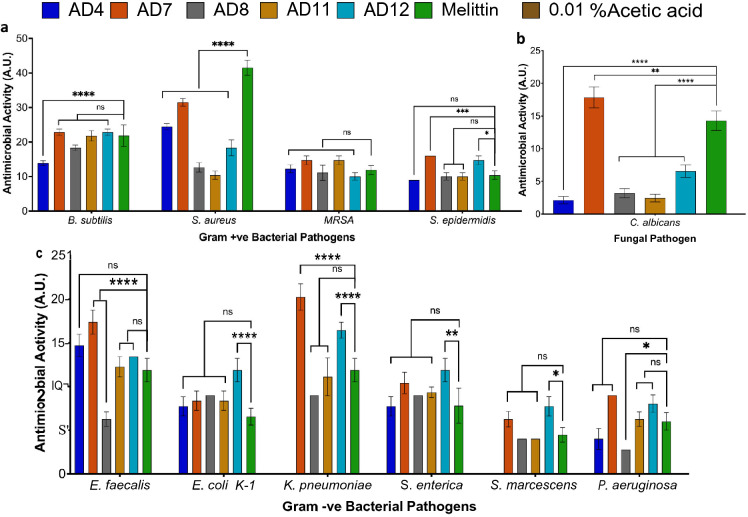
Screening the antimicrobial activities spectra of the selected cryptides. The antimicrobial activity spectra of the selected cryptides were screened by radial diffusion assay. The observed activities were correlated with the diameter of the inhibition zones and expressed in absolute units (A.U.). (**a**) the measured antibacterial activity against the chosen Gram-positive pathogens, (**b**) The measured antifungal activity against *C. albicans*, and (**c**) the measured antibacterial activity against the chosen Gram-negative pathogens. All the observed activities were compared to the corresponding positive control by one-way ANOVA test, (****) highly significant, P-value ≤ 0.0001; (***) significant, P-value ≤ 0.0002; (**) marginally significant, P-value ≤ 0.0021; (*) low significant, P-value ≤ 0.0332; *ns* not significant.

The antimicrobial activity spectra of the chemically synthesised cryptides were screened against different microbial pathogens by radial diffusion assay. The antimicrobial activity correlated with the diameter of the inhibition zone. For these experiments, melittin and the vehicle control (0.01% acetic acid) were used as the positive and negative controls, respectively. No inhibition zones were identified around the negative control-treated wells, while most of the tested cryptides against Gram-positive pathogens were as active as melittin without significant differences among the measured activities (Fig. 2, Fig. S.2). Lower activity was reported for all the tested cryptides against *S. aureus* as well as AD4 against *B. subtilis*. Interestingly, AD7 and AD12 showed significantly higher activity than melittin against *S. epidermidis*. Likewise, most of the tested cryptides against Gram-negative pathogens were as active as melittin without significant differences among the measured activities (Fig. 2c). The only observed lower activity was for AD8 against *E. faecalis* and *P. aeruginosa*. AD7 showed higher activity against *E. faecalis* and *K. pneumonia* while AD12 showed the same results against *E. coli* and *K. pneumonia* as well as *S. enterica*. On the other hand, all the tested cryptides showed significantly low antifungal activity when compared with melittin, while AD7 was the only cryptide with higher antifungal activity (Fig. 2b, Table S.3).

For quantitative assessment, the micro-broth dilution technique was used to determine the minimum inhibitory concentration (MIC), minimum bactericidal concentration (MBC), and minimum fungicidal concentration (MFC) values against the tested pathogens (Table 2). AD7 showed MIC values of 0.39 ~ 6.25 µM, MBC values of 0.39 ~ 25 µM, and MFC of 6.25 µM, against the different tested pathogens. The measured values of AD7 against each pathogen were lower or equal to the corresponding values of other cryptides. AD7 was the only cryptide that showed detectable MIC and MBC values against *V. parahaemolyticus* within the tested concentration range. Moreover, the observed MBC/MFC values of AD7 were equal to the corresponding MIC values except against *P. aeruginosa* and *C. albicans.* Regardless of the undetected MIC and MBC values of AD8 and AD12 against *V. parahaemolyticus*, AD8 showed MIC values of 0.78 ~ 25 µM, MBC values of 0.78 ~ 50 µM and MFC value of 6.25 µM. These values were lower or equal to the corresponding values of AD12 against each pathogen, except *P. aeruginosa,* which showed MIC and MBC values of 0.78 ~ 12.5 and an MFC value of 12.5. AD11 didn’t exhibit activity against *V. parahaemolyticus,* while AD4’s MICs and MBCs were undetected against *P. aeruginosa* and *V. parahaemolyticus* within the tested concentration range.

**Table 2:** MIC, MBC and MFC of the selected cryptides.

Cryptides	Gram-positive				Gram-negative						Fungus	
	*B. subtilis*		MRSA		*S. enterica*		*P. aeruginosa*		*V. parahaemolyticus*		*C. albicans*	
	MIC (µM)	MBC (µM)	MIC (µM)	MBC (µM)	MIC (µM)	MBC (µM)	MIC (µM)	MBC (µM)	MIC (µM)	MBC (µM)	MIC (µM)	MFC (µM)
AD4	1.56	1.56	6.25	12.5	3.12	3.12	> 50	> 50	> 50	> 50	25	25
AD7	0.39	0.39	3.12	3.12	0.78	0.78	6.25	25	3.12	3.12	1.56	6.25
AD8	0.78	0.78	3.12	12.5	0.78	0.78	25	50	> 50	> 50	6.25	6.25
AD11	0.78	0.78	6.25	12.5	1.56	1.56	25	> 50	NA	NA	25	25
AD12	0.78	0.78	6.25	25	0.78	0.78	12.5	25	> 50	> 50	12.5	12.5

MIC values were determined by the micro-broth dilution technique. Then, 20 µl of each concentration without optically detectable growth was incubated on agar plates for 18 h to determine the MBC and MFC values of each cryptide.

*MIC* minimum inhibitory concentration, *MBC* minimum bactericidal concentration, *MFC* minimum fungicidal concentration, *NA* No activity detected.

### Antibiofilm activities 

**Figure 3:**
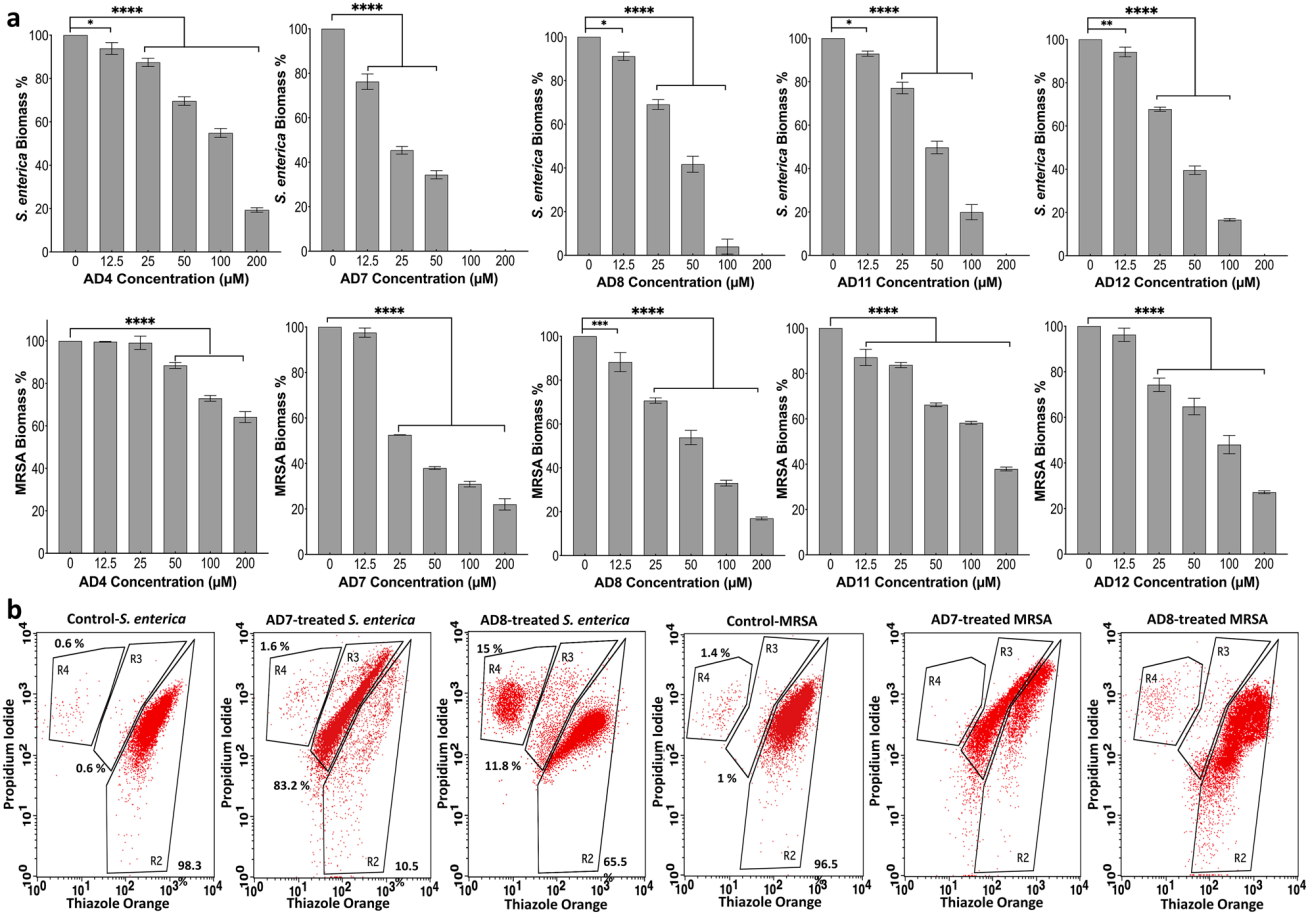
Antibiofilm activity and microbial membrane integrity. (**a**) the measured biomass of *S. enterica* and MRSA in response to treatment with the selected cryptides by microtiter plate assay. The observed biomass percentage in response to each treatment was compared to the corresponding negative control by one-way ANOVA test, (****) Highly significant, P-value ≤ 0.0001; (***) Significant, P-value ≤ 0.0002; (**) Marginally significant, P-value ≤ 0.0021; (*) Low significant, P-value ≤ 0.0332. (**b**) The effect of AD7 and AD8 on the integrity of gram-negative (*S. enterica*) and Gram-positive (MRSA) microbial membranes by FACS. R2, cells with intact membranes; R3, cells with compromised membranes and R4, cells with totally damaged membranes. The machine was adjusted to acquire 1 × 10^4^ cells.

Microbial biofilms are a significant risk factor for persistent biofilm-related chronic infections, implantation failure of medical devices, and decontamination failure of surgical instruments and related surfaces^21^. Therefore, we tested the ability of the selected cryptides to eradicate mature bacterial biofilms. All the selected cryptides showed significant antibiofilm activities against both *S. enterica* and MRSA compared to negative controls, as shown in Fig. 3a. AD7 showed significant activity against *S. enterica* starting from the lowest concentration (12.5 µM) with 23.79% biofilm eradication. The observed minimal biofilm eradication concentration (MBEC) of AD7 was 100 µM while AD8, AD11, and AD12 showed MBEC of 200 µM. The maximum biofilm eradication shown by AD4 against *S. enterica* was 80.63% at 200 µM. Although none of the selected cryptides showed complete eradication of MRSA mature biofilm, they showed significant activity at the highest tested concentration (200 µM). AD8 showed the highest activity with 83% biofilm eradication, followed by AD7, AD12, AD11, and AD4 with 78%, 73%, 62%, and 35.8% biofilm eradication, respectively (Fig. 3a, Table S.4).

### Effects of cryptides on membrane permeabilisation and cell viability 

Cryptides AD7 and AD8 were further selected to evaluate their ability to permeate the microbial cells, as it has been previously described that the mode of action of cryptides may be via membranolytic or non-membranolytic pathways^22^. Briefly, high counts of *S. enterica* and MRSA (5 X 10^5^ cells) were treated with a high concentration of AD7 and AD8 peptides (50 µM) for 3 h. Treated microbial cells were incubated with a dye combination (thiazole orange; TO that stains cells with intact membrane structures, and propidium iodide; PI that stains dead cells with damaged cell membranes. Staining with both dyes is indicative of injured cell membranes)^23^. Vehicle control-treated *S. enterica* and MRSA had intact cell membranes (panels 1 and 4, Fig. 3b), as indicated by the high levels of staining with TO (98.3% and 96.5%, respectively). AD7 treatment mainly caused cellular injury to *S. enterica* cells (83.2%, panel 2 Fig. 3b), but its effects on cellular membrane injury were less pronounced in MRSA (54.3%, panel 5 Fig. 3b). AD8-treated *S. enterica* showed that most of the cells (65.5%) had intact cell membranes and were viable, while the remaining were either dead (15%) or had compromised membranes (11.8%; panel 3, Fig. 3b). A similar effect pattern was noted when AD8 was used to treat MRSA, albeit with more viable bacterial cells (88.4%), indicating a less pronounced effect (panel 6, Fig. 3b).

### Anti-cancer activity of the selected cryptides

**Figure 4:**
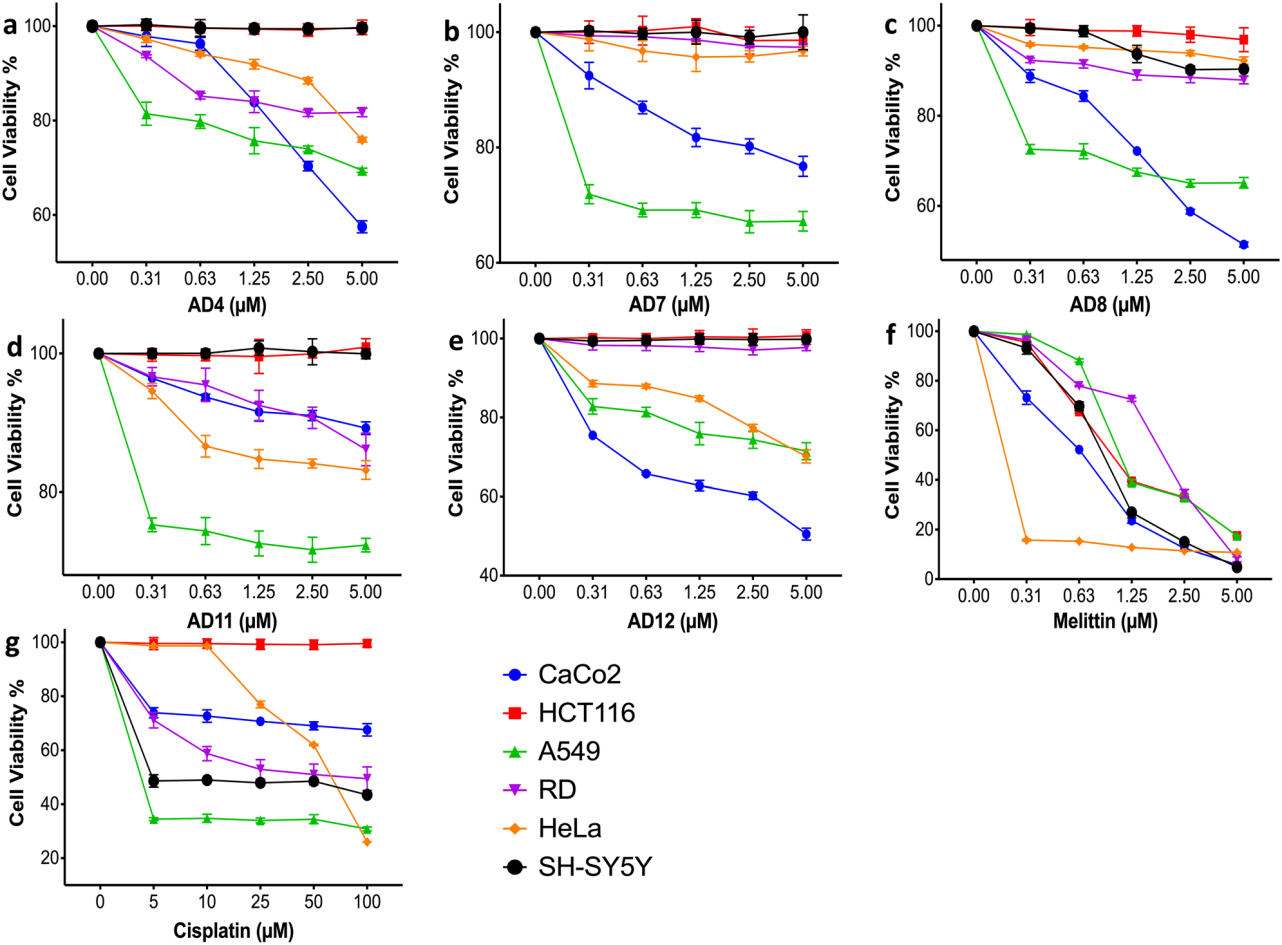
The anti-cancer activity of the selected cryptides. Graphs a-g show the measured cell viability by MTT assay, of the tested cancer cells in response to 24-h treatment in serum-free medium at 37 °C and 5% CO_2_ with varying concentrations of cryptides and two positive controls, melittin and cisplatin.

The anti-cancer activities of the cryptides were evaluated by calculating the viability percentages of the tested cells in response to treatment with different concentrations of each cryptide for 24 h in a serum-free medium. First, the effect of serum deprivation was tested on the selected cell lines before treatment. All cell lines studied could withstand the serum starvation for 24 h without significant decline in their cell viabilities, supplementary Fig. S.3. Hence, the effect of serum deprivation on the cell lines is neglectable. However, the selected cryptides (AD4, AD7, AD8, AD11, and AD12) showed anti-cancer activities against most of the tested cancer cell lines in the serum-free medium (Fig. 4a–e), similar to melittin and cisplatin, which were used as positive controls in this study (Fig. 4f and g). The highest and lowest calculated IC_50_ values were reported for AD12 against rhabdomyosarcoma (RD) and human colorectal adenocarcinoma (Caco-2) as 158.85 µM and 4.35 µM, respectively (Table 3). AD8 was the only active cryptide against neuroblastoma cells (SH-SY5Y) with a calculated IC_50_ of 23.3 µM, while human colorectal carcinoma cells (HCT 116) were resistant to all the tested cryptides and cisplatin (Table 3).

**Table 3:** IC_50_ of the selected cryptides against the tested cancer cell lines.

Compound	IC_50_ (µM)					
	Caco-2	HCT 116	A459	HeLa	SH-SY5Y	RD
AD4	5.4 ± 0.7	NA	8.8 ± 1.1	10.8 ± 0.3	NA	15 ± 0.8
AD7	11.2 ± 0.7	NA	8.5 ± 0.6	101.5 ± 0.9	NA	97.8 ± 0.2
AD8	4.4 ± 0.5	NA	7.3 ± 0.5	42.6 ± 0.3	23.3 ± 0.7	27.2 ± 0.4
AD11	26.8 ± 0.4	NA	11.1 ± 0.7	16.8 ± 0.6	NA	19.2 ± 0.9
AD12	4.3 ± 0.6	NA	9.4 ± 1	8.5 ± 0.5	NA	158.8 ± 0.5
Melittin	1.2 ± 0.9	2.1 ± 0.2	2.3 ± 0.6	0.9 ± 0.1	1.7 ± 1	2.4 ± 0.5
Cisplatin	174.3 ± 0.8	NA	15.3 ± 0.6	63.6 ± 0.4	54.4 ± 0.8	74 ± 1.2

The cells were treated with different concentrations of each desired compound for 24 h in a serum-free medium. The cell viabilities in response to each treatment were measured by MTT assay, by which the death rates and IC_50_ values were calculated.

*IC*_50_, inhibitory concentration, *NA* no activity detected.

### Safety assessment of the selected cryptides

**Figure 5:**
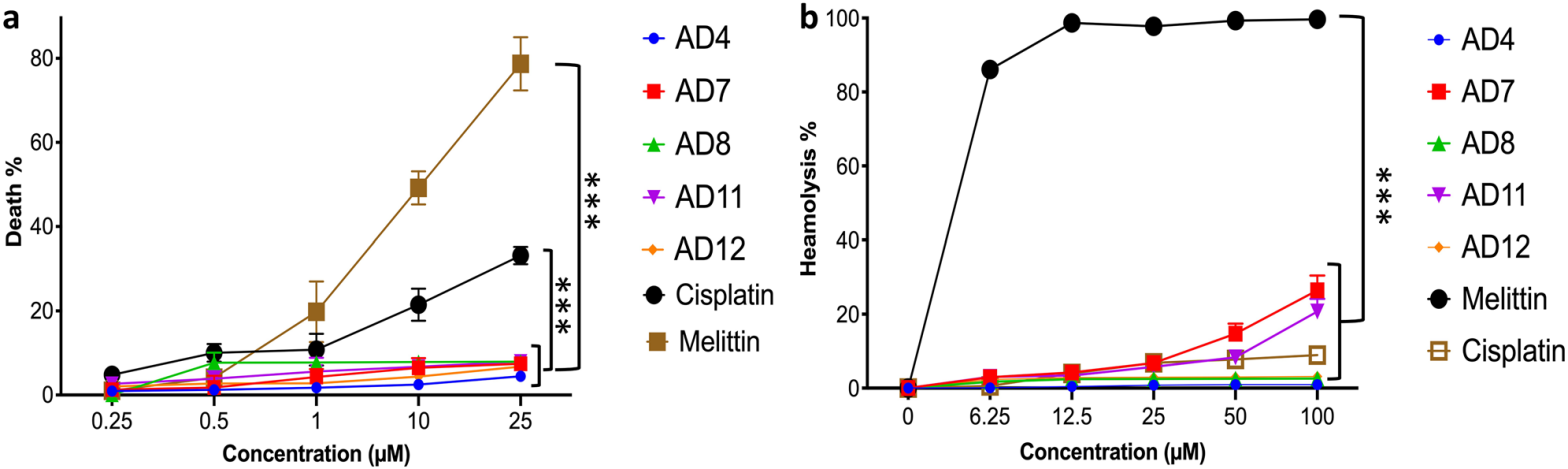
The cytotoxic and hemolytic effects of the selected cryptides against HEK 293 and human RBCs. (**a**) the measured viabilities of HEK 293 by MTT assay in response to treatment with different concentrations of each cryptide for 24 h in serum-free media at 37 °C and 5% Co_2_, (**b**) the hemolytic effects of the selected cryptides on human RBCs by treating 2% RBCs with different concentrations of each cryptide for 2 h at 37 °C. The treated cells were centrifuged, and the supernatants were measured at 450 nm. Melittin and cisplatin were used as positive controls while 0.1% TritonX-100 was used to determine the maximum hemolysis activity. All the observed effects were compared to positive controls by two-way ANOVA test, (****) highly significant, P-value ≤ 0.0001.

Since the toxicity effect of any drug on normal human cells significantly impacts drug development, regardless of the effectiveness of this drug^24^, it was essential to evaluate the harmful effects of the selected cryptides on healthy human cells. Therefore, we tested their effects against normal human cells HEK-293 and red blood cells (RBCs). As shown in Fig. 5a, AD4, AD7, AD8, AD11, and AD12 showed a minor, dose-dependent toxicity pattern on HEK-293 cells with less than 10% cell death at the highest tested concentration (25 µM). Accordingly, they showed very low cytotoxic effects compared with cisplatin and melittin, resulting in 33% and 78.7% cell death when treated at 25 µM, respectively.

Moreover, the tested cryptides showed mild effects on human RBCs up to 100 µM, with less than 10% hemolysis, except AD7 and AD11, which started to show moderate hemolysis from 50 µM with maximum hemolysis rates of 26.5% and 20.7% at 100 µM, respectively. Melittin showed almost 100% hemolysis starting from 12.5 µM, while cisplatin showed less than 10% at the highest tested concentration (100 µM) (Fig. 5b).

## Discussion

Cancer and infectious diseases are both major causes of morbidity and mortality^25^. In some cases, microbial infections may also occasionally lead to cancers later in life. For example, *Salmonella enteritidis* and *Salmonella typhi* infections have been linked to colon and gall bladder cancer development, respectively^26^. Other instances include hepatocellular carcinoma, which is brought on by the hepatitis C virus infection^27^, cervical cancer, where 95% of its cases are caused by sexually transmitted human papillomavirus (HPVs)^28^, and lung cancer, which may be caused by bacterial infections^29^. On the other hand, immunodeficiency is strongly associated with the onset of cancer, which increases the risk of infection-related death among cancer patients exposed to MDR pathogens^30^. Many antibiotic-resistant cancer-associated microbial infections were reported, including *Klebsiella* spp., *Pseudomonas* spp., and *E. coli,* which have significant rates of antibiotic resistance^31^.

Additionally, the rapid and ongoing genetic mutations in MDR bacteria and cancer cells undermine the effectiveness of currently available antibiotics and cancer chemotherapies^31^. Therefore, there is an urgent need to develop novel and sustainable antimicrobial and anti-cancer agents to manage these conditions. Cationic cryptides may serve as suitable candidates to meet this demand as their efficacy is not affected by the alteration caused by genetic mutations of microbes and cancer cells. In our study, we demonstrate a pipeline to mine available RNA-seq data to identify novel cryptides with potential desired activities and to screen for their biological activities using in vitro-based assays. Such an approach would allow for the rapid identification and development of these much-needed drugs systematically and sustainably as it minimises research time and cost and avoids unnecessary animal usage for research compared to the traditional methods of bioactive compound screening.

In this study, we could computationally separate cryptides from their precursors depending on the preferences of specific amino acids for antibacterial activity, such as glycine, leucine, lysine, cysteine, and arginine, using AntiBP online server^32^. We also predicted their potential antimicrobial and anti-cancer activities, toxicity, hemolysis activity, and cell penetration ability by using online tools that utilise machine learning algorithms based on the presence or absence of specific structures/motifs, referring to different databases of previously studied peptides with similar effects. As a result, various Gram-positive and negative bacteria and fungi were susceptible to our chemically synthesised cryptides, albeit at different activity levels.

Of the five cryptides evaluated, AD7 and AD12 showed significantly greater antimicrobial activities against different Gram-positive and Gram-negative pathogens as well as AD7 against *C. albicans*, compared to the control groups. Furthermore, AD7 had MIC and MBC/MFC values that were lower or equivalent to other cryptides and showed the only detected values against *V. parahaemolyticus,* the pathogen *P. vannamei* was challenged with to originate the RNA-seq dataset that has been selected in our study. This observation would indicate the validity of our in silico approach for the identification of bioactive cryptides from the transcriptomic data of living organisms. The MIC values of AD8 and AD12 were similar, except against *P. aeruginosa* where the activity of AD12 was superior to AD8. However, the situation was reversed for *C. albicans*, where AD8 had a lower MIC than AD12.

The bioactivity of these cryptides correlated to their net charges and hydrophobicity rates, which have been previously shown to initiate the attraction of the cationic cryptides to the negatively charged microbial membranes^33^. In this study, the superior antimicrobial and anticancer activities of AD7 and AD12 could be attributed to their higher net positive charges (+ 5 and + 4, respectively) and hydrophobicity (53%) compared to the other peptides. Interestingly, despite possessing a greater net positive charge than AD12 and AD8, AD4 displayed the lowest antimicrobial efficacy against the microorganisms examined. This could be due to its low hydrophobicity (26%), which determines the peptide’s ability to penetrate the cell membrane and its membranolytic characteristics. These attributes are also influenced by other physical–chemical properties such as conformation, net charge, and amphipathicity, as reported in previous research studies^34^.

Compared to their planktonic growth, bacterial biofilms may withstand harsh environmental conditions such as antimicrobial agents 10–1000-fold^34,35^. Cationic cryptides represent a secure and less complicated strategy against microbial biofilms, either alone or combined with antibiotics^36^, compared to the antibiotics combination therapy that exacerbates the problem of antibiotic resistance by accelerating the natural selection of antibiotic-resistant organisms^37^ and antibiotics-viral phages combination therapy due to the difficulty of predicting eligible combinations that promote biofilm eradication^38^. Indeed, ll-37 was reported as a potential antimicrobial and antibiofilm agent that could be an alternative to conventional antibiotics^39^. In the present study, the tested cryptides showed an eradication ability of Gram-positive and negative mature biofilms at very high concentrations when compared with their potency to eliminate the planktonic cells of the same pathogens, which is consistent with previous findings^40^. The observed reduced sensitivity of MRSA to the tested cryptides could be attributed to the effect of the positively charged polysaccharide intracellular adhesin (PIA) that protects *S. aureus* and *S. epidermidis* from cationic cryptides via electrostatic repulsion^41^.

We evaluated the mode of action of AD7 and AD8 in their antimicrobial activity using a FACs-based assay. According to our findings, AD7 caused membrane injury in both *S. enterica* (Gram-negative) and MRSA (Gram-positive) bacteria. AD8, however, seemed to be less potent, with a lesser number of dead and compromised cells in the time-controlled assay. This data is congruent with the MIC/MBC values of the same bacteria-cryptide combination. In a previous publication, a cryptide originating from a rattlesnake venom peptide, Ctn 15–34 was demonstrated to exert bactericidal effects against two antibiotic-resistant Gram-negative bacterial species^42^. In contrast, another study that treated Gram-negative *E. coli* with high concentrations (2X MIC) of bacterial cationic non-ribosomal peptides did not show significant bacterial membrane damage^43^. It is possible that cryptides may assume different mechanisms of action based on their physical and chemical properties. Hence, more studies that involve time- and dose-dependent assays are needed to get better insights into this.

All the selected cryptides demonstrated anti-cancer effects against most of the tested cancer cell lines. The observed resistance of HCT 116 cells to the tested cryptides could be attributed to several factors, including high membrane rigidity due to higher cholesterol levels^44^ and genomic aberrations that may contribute to drug resistance^45^. The resistance of the SH-SY5Y cells towards four of the five peptides tested may also be due to its relatively higher mitochondrial membrane potential and electrical membrane activity, inhibiting the attraction between the cationic cryptides and cellular and mitochondrial membranes^46^. These observations highlight the importance of evaluating improved drug efficacy by combining both peptides and existing registered drugs that may improve the delivery system of anti-cancer drugs. For example, the combination of atorvastatin and celecoxib^47^ or angiopep-2 and doxorubicin^48^ resulted in an improved cancer cell targeting efficacy compared to the drug alone.

Regarding safety, our selected cryptides exhibited a negligible dose-dependent cytotoxic effect against the normal human cells, recording less than 10% cell death and neglectable hemolytic effect against human RBCs, demonstrating that our cryptides meet the international safety standards^49^.

Overall, our results support the theory that cationic peptides may exhibit dual activities against microorganisms and cancer cells due to their propensity to selectively attract and non-specifically interact with negatively charged biological membranes via electrostatic and hydrophobic forces that are stronger than the hydrophobic attraction between them and healthy cells^25^.

This research mainly concentrates on the ability of cryptides to combat microorganisms and cancer cells; however, the scope of their potential is much broader. While this study has explored their effectiveness against microorganisms and cancer cells, they could also prove beneficial against parasites and enveloped viruses. Additionally, cryptides have previously been demonstrated to have immunomodulatory, antioxidant, anti-inflammatory, wound-healing, cardioprotective, and libido-enhancing properties^50,51^. The future direction of our research will include investigating the mechanisms of action of cryptides, uncovering their other potential biological activities, and clinical trials to test for their efficacy in practice.

## Conclusion

The study of cryptides and their biological activities is in its infancy. We believe this field could accelerate the discovery of novel peptide-based molecules and expand their therapeutic potential against MDR microbial and cancer cells. Our study proposes a new strategy for identifying natural cryptides from living organisms by computational analysis of the already produced transcriptomic datasets and predicting their potential bioactivities based on the sequence-structure relationship and the similarity with conserved signatures. The obtained in vitro results of the selected cryptides demonstrate their potency as antimicrobial and anti-cancer agents with negligible cytotoxicity toward healthy cells. Thus, the current work demonstrates a proof-of-concept for the rapid and sustainable mining and validation of peptide-based therapeutic agents, paving the way for discovering novel drugs against multidrug-resistant microbes and cancer cells.

## Methods

### Computational identification and characterisation

The NCBI-SRA database was screened for paired-end RNA-seq data for *P. vannamei*, challenged with a bacterial infection. The chosen data set (SRP126153) was retrieved from NCBI-SRA and was subjected to a quality control check by the FastQC application (v 0.11.9)^52^. Based on the obtained QC reports, the data was cleaned from sequencer adaptors, PCR primers, and any read with low length and/or quality by the Trimmomatic software (v 0.39)^53^. The trimmed data were used for de novo transcriptome assembly by Trinity software (v 2.13.2), followed by extraction of the longest isoforms from the transcriptome assembly output using the Transcoder utility^54^. For functional annotation, the extracted longest isoforms were used as query sequences for homology search by the standalone version of BLASTx against a customised manually prepared database that compiled all up-to-date identified AMPs sequences in kingdom Animalia, at E-value cut-off was 1e-4, following the instructions given in the user manual of the NCBI-BLAST command-line application^55^. The gene-encoded AMPs were then extracted from the BLASTx output file and filtered using (ALF, Crustin, Penaeidin and Stylicin) as keywords. Then gene-encoded AMPs precursors were primarily selected based on their novelty, without previous publication. The bioactive encrypted segment within each of them, with the highest score, was identified by the online server AntiBP^32^.

ProtParam, a tool of ExPasy was used to compute physical and chemical parameters of the detected cryptides, such as molecular weight, hydrophobicity, and theoretical isoelectric points^56^. The identified cryptides were characterised as antimicrobial peptides (AMPs) or non-antimicrobial peptides (nonAMPs), and their net charges at pH = 7 were predicted by ADP3 database server^57^. ToxinPred online server was used to predict the potential toxic effect of cryptides against normal human cells^58^. The prediction of potential hemolysis activities, potential anti-cancer activities, and potential cell penetration abilities was done using the HemoPI server^59^, AntiCP^60^ and BChemRF-CPPred^61^ respectively. Finally, cryptides were selected based on their net positive charge and potential cell penetration ability and subjected to the PepFold3 server for de novo 3D structural prediction^62^. The predicted 3D structures and the electrostatic potential on their surfaces were visualised by ChimeraX software (v 1.3)^63^.

### Peptide synthesis

The selected cryptides were synthesised by the solid-phase method and purified by HPLC followed by mass spectrometry analysis to confirm the purity of the final product at Apical Scientific Co. (Malaysia). The received lyophilised peptides were dissolved into 0.01% acetic acid (Sigma-Aldrich, A6283) and stored at − 20 °C for further usage.

### Microbial strains and cultural conditions 

The microbial strains used in this study were *Bacillus subtilis* (ATCC, 11774), *Candida albicans* (TIMM, 1768), *Enterococcus faecalis* (ATCC, 33186), *Escherichia coli* (ATCC, 11775), *Klebsiella pneumoniae* (ATCC, 13883), Methicillin-resistant *Staphylococcus aureus* (ATCC, BAA-41), *Pseudomonas aeruginosa* (ATCC, 10145), *Salmonella enterica* (ATCC, 14028), *Serratia marcescens* (ATCC, 13880), *Staphylococcus aureus* (ATCC, 25923) and *Vibrio parahaemolyticus* (ATCC, 17802). All microbial strains were cultivated overnight at 37 °C and 200 rpm, then subcultured and incubated for 3 h under the same incubation conditions to obtain the mid-logarithmic growth phase. The cultivated cells were washed twice with 10 mM Tris–HCl containing 5 mM D-glucose at pH 7.4 and suspended in the same buffer to be used in different assays, as previously described^64^.

### Antimicrobial assays

The antimicrobial activity spectra of the selected cryptides were screened against all the mentioned microbial pathogens, except *V. parahaemolyticus,* by radial diffusion assay as previously described^64^, with slight modifications. We aimed to test different categories of pathogens on the WHO global priority due to their growing resistance to the available antibiotics such as *P. aeruginosa*, *E. coli* and *K. pneumoniae* in the critical category and *Salmonella* ssp. and *S. aureus* in the high category^65^. Briefly, a total of 4 × 10^6^ CFU of each pathogen was inoculated in 10 mL of an underlay agarose gel [0.03% w/v tryptone soy broth (TSB) medium, 1% w/v agarose and 0.02% v/v Tween 20 in 10 mM Tris–HCl] that was then poured into a 100-mm petri dish. After agarose solidification, 3 mm-diameter wells were punched by a sterile cork borer and 5 µl of 500 µM stock solution from each cryptide was added to each well. Melittin and acidified water were used as positive and negative controls, respectively. After 3 h incubation at 37 °C, the underlay gel was covered with 10 mL of nutrient-rich overlay [6% w/v TSB and 1% w/v agarose in 10 mM Tris–HCl]. The plates were then incubated for 18 h, and the antimicrobial activities were measured in the absolute unit (AU), as previously described^66^.

As a conclusive quantitative assessment, broth microtiter assay was used to determine the MIC values of each cryptide against *B. subtilis*, MRSA, *S. enterica*, *P. aeruginosa, V. parahaemolyticus* and *C. albicans* by following the guidelines of the Clinical and Laboratory Standards Institute (CLSI)^67^, with slight modifications. Eighty microliters of 5 × 10^4^ CFU/ml of each pathogen were dispensed into a 96-well polypropylene microtiter plate. The wells were then treated with 20 µl of serially diluted cryptides for 3 h at 37 °C. Gentamicin was used for positive controls at 0.2 mg/ml while an equivalent volume of acidified water was used for negative controls. The plates were then topped up with a final concentration of 1 × Mueller Hinton Broth (MHB) and incubated for 18 h under the same incubation conditions. MIC was detected as the lowest concentration of each cryptide that prevents the optically detectable microbial growth at OD = 600 nm. Then 20 µl of each concentration, starting from MIC, were spotted and incubated on Mueller Hinton Agar (MBA) or Sabouraud 4% Dextrose Agar at 37 °C for 18 h to determine the MBC and MFC values as the lowest concentration of each cryptide that prevented the microbial growth on the agar plates^66^.

### Antibiofilm assay 

The antibiofilm activities of the selected cryptides against *S. enterica* and MRSA mature biofilms were examined by microtiter plate assay as previously described^68^, with slight modification. During the mid-logarithmic phase, *S. enterica* and MRSA were suspended at the density of 1.7 × 10^8^ CFU/ml in MHB. Then 100 µl of each bacterial suspension was dispensed into a vinyl 96 U-shaped-well plate and incubated overnight at 37 °C. The mature biofilms were washed with 10 mM Tris–HCl containing 5 mM D-glucose at pH 7.4 and treated for 8 h with 100 µl of two-fold serially diluted cryptides, under the same incubation conditions. A sterile MHB medium was used as the positive control while an equivalent volume of acidified water was used to treat the negative controls. The treated biofilms were washed twice with the same washing buffer and stained with 0.1% crystal violet for 15 min followed by rinsing with sterile distilled water twice and then dried for 30 min at 37 °C. The stain was dissolved with 33% acetic acid and transferred to an optically clear 96-well flat bottom plate which was then measured at 600 nm.

### Microbial membrane integrity

Two cryptides were chosen to check their effect on microbial cell membranes by FACS. For this purpose, we used a cell viability kit (BD Pharmingen, 349,483), following the manufacturer’s instructions^69^. A total count of 5 × 10^5^ cells were treated with 50 µM of each cryptide for 3 h at 37 °C. Equivalent volumes of acidified water were added to negative controls. The treated cells were then centrifuged at 3700 g for 5 min and resuspended into 500 µl of staining buffer [1 × PBS containing 0.2% pluronic F-68 and 1 mM EDTA], in a 5 ml falcon tube. The cells were stained with 5 µl of TO and PI for 5 min in the dark at room temperature (RT). They were then assessed by a flow cytometer within 1 h of staining at a slow rate flow and cell count of 1 × 10^4^ cells.

### Mammalian cell culture and maintenance

The used mammalian cell lines in our study were colorectal adenocarcinoma (Caco2, ATCC-HTB-37), colorectal carcinoma (HCT 116, ATCC-CCL-247), lung carcinoma (A549, ATCC- CCL-185), cervical cancer cells (HeLa, ATCC-CCL-2), neuroblastoma (SH-SY5Y, ATCC- CRL-2266), rhabdomyosarcoma (RD, ATCC- CCL-136) and human embryonic kidney cells (HEK 293, ATCC- CRL-1573). All the cells were maintained into Dulbecco’s Modified Eagle’s Medium (DMEM) supplemented with 10% heat-inactivated fetal bovine serum, 2 mM l-Glutamine, 100 U/ml penicillin–streptomycin (Pen/Strep) at 37 °C in 5% CO_2_ humidified incubator.

### Cytotoxicity assessment

First, the cytotoxic effect of 24 h serum deprivation on all the selected cell lines was evaluated by MTT assay, following standard protocols^70^. Briefly, cells were seeded and maintained at 37 °C and 5% CO_2_ in 24-well plates until they reached 75–80% confluency (zero time). Following washing twice with 1X PBS, cells were incubated in a serum-free medium for 24 h, under the same incubation conditions. The cell viability percentages were calculated and compared to zero time as 100% viability to nullify any possible effects of serum deprivation. Thence, the cell viability percentage of each cell line in response to 24 h treatment with different concentrations of each cryptide in serum-free medium was calculated. Melittin and cisplatin were used as positive controls, while 0.01% acetic acid was used as the negative control. All the measured cell viability percentages were normalised against the corresponding negative controls.

### Ethical approval and human RBC preparation

All methods were carried out in accordance with the Declaration of Helsinki, and the Research Ethics Committee approved all experimental protocols at Sunway University (approval no. PGSUREC2020/005). A human blood sample was collected from a healthy 27 years old female for this assay upon obtaining informed consent. The fresh blood sample was centrifuged at 3700 *g* for 5 min at RT. The supernatant (serum) was removed and red blood cells (RBCs) were washed three times with 1X PBS and diluted to a final concentration of 2% (PBS—137 mM NaCl, 2.7 mM KCl, 10 mM Na_2_HPO_4_, 1.76 mM KH_2_PO_4_). Cells were stored at 4 °C, and used within 5 h.

### Hemolytic assay

The hemolytic activities of the identified cryptides were evaluated as previously described^71^. The prepared RBCs were treated with different peptide concentrations for 2 h at 37 °C. The treated RBCs were centrifuged, under the same centrifugation conditions, and the supernatants were transferred to a 96-well plate and measured at 450 nm. Melittin and cisplatin were used as positive controls, negative controls were treated with an equal volume of the vehicle, and 0.1% TritonX-100 was used to determine the maximum hemolysis activity.

### Statistical analysis

Data are expressed as mean ± standard deviation (SD) from 3 separate experiments in triplicate samples. Statistical analysis was performed using ANOVA and Tukey-test to enable pairwise comparison with GraphPad (V 9.4.1).

### Supplementary Information

Supplementary Information.

## Data Availability

The described data in this manuscript are included in the article's tables, supplementary information and supplementary data.
