# Supplementary material for: Novel cationic cryptides in Penaeus vannamei demonstrate antimicrobial and anti-cancer activities
Source: Sci Rep. 2023 Sep 6;13:14673. doi: 10.1038/s41598-023-41581-9 (PMC10482825; doi:10.1038/s41598-023-41581-9)
Supplement: Supplementary file 1 — Supplementary Information. [file 41598_2023_41581_MOESM1_ESM.pdf]

Novel cationic cryptides in *Penaeus vannamei* demonstrate antimicrobial and anti-cancer activities

Amr Adel Ahmed Abd El-Aal[1,2], Fairen Angelin Jayakumar[1], Chandrajit Lahiri[1,3#], Kuan Onn Tan[1] and Kavita Reginald[1*]

[1]Department of Biological Sciences, School of Medical and Life Sciences, Sunway University, Bandar Sunway 47500, Selangor, Malaysia

[2]National Institute of Oceanography and Fisheries (NIOF), 84511, Egypt.

[3]Department of Biotechnology, Atmiya University, Rajkot, 360005 Gujarat, India.
[#]Present address

*Corresponding author:

Assoc. Prof. Dr. Kavita Reginald, Department of Biological Sciences, School of Medical and Life Sciences, Sunway University, Bandar Sunway 47500, Selangor, Malaysia

Tel. +60-374918622; Fax +60-3 5635 8633; E-mail: kavitar@sunway.edu.my

```
###################################
## Counts of transcripts, etc.
###################################
Total trinity 'genes':    85875
Total trinity transcripts:         120619
Percent GC: 43.10
############################################
Stats based on ALL transcript contigs:
############################################
Contig N10: 7691
Contig N20: 5768
Contig N30: 4547
Contig N40: 3614
Contig N50: 2781

Median contig length: 469
Average contig: 1200.23
Total assembled bases: 144770290
##########################################################
## Stats based on ONLY LONGEST ISOFORM per 'GENE':
##########################################################
Contig N10: 6445
Contig N20: 4552
Contig N30: 3328
Contig N40: 2425
Contig N50: 1712

Median contig length: 374
Average contig: 821.25
Total assembled bases: 70524917
```

Figure S.1 Trinity assembly statistics. Only paired output files from data processing were subjected to *de novo* transcriptome assembly by the Trinity assembler pipeline that was executed by a single Perl script called Trinity pl.

Table S.1 ORFs and Protein sequences of the selected gene-encoded AMPs (precursors). The DNA sequences of the selected gene-encoded AMPs were extracted from the Trinity output file and subjected to the online open reading frame finder tool to get their ORFs and the translated protein sequences.

Trinity ID	Nucleic acid sequence	Strand	Length (bp)	Amino acid sequence	Length (aa)
DN1899	**ATG**CAGCAGGTACTGTATTTGACATTACTATGGGTGTGCCTTGGGAC<br>TGGCGTGAGGAGCTCGGCGATCGACGTCCCAGAAGCGACGCCGCCC<br>TCCTACTCCAACTGCGAATTCTGGTGCAAAACGCCAGATAACGCGTT<br>TTACTGCTGCGATAATGACGGCGAGACAGTCACTCCGGAAGAGCCTG<br>AGCACGATGGCGAGTGTCCCCAGGTACGGAGGGAATGCCCCGGGAT<br>ATACAAGAATCTTCCCAGTACATTGCCAGACTTCGACATTTATGGAG<br>TGCCTTATCCTGATGACCCCAAGTTCTGCGCCCATGACAGTGTCTGCA<br>GCGCCTGGGAAAAGTGCTGTTTCGACCGCTGCTTCGAACGCCATGTC<br>TGCAAGTTCGCGTACAGTACTGACACAGATGTAGAGTATTCGGAGAG<br>TGCATTCCGGGACGGAGACATTGTCCAAGAAGCCACCTATCTATTAG<br>GA*TGA*	(-)	474	MQQVLYLTLLWVCLGT<br>GVRSSAIDVPEATPPSYS<br>NCEFWCKTPDNAFYCC<br>DNDGETVTPEEPEHDGE<br>CPQVRRECPGIYKNLPS<br>TLPDFDIYGVPYPDDPK<br>FCAHDSVCSAWEKCCF<br>DRCFERHVCKFAYSTDT<br>DVEYSESAFRDGDIVQE<br>ATYLLG	157
DN35987	**ATG**CGCGTGATCGTCCTTGCACTCCTGGTGGCGGCGTCCTCTGCCTG<br>CCGCTATTACTGCAAAACTTTAAGTGATGAAACCTACTGTTGCGATG<br>GCGGGAAAGATTACAACCCTCCCGAATACCACGACGGAAATTGCCC<br>GAAGGTTCGCAGCTTCTGCCCGCGAATCGAGGGTGGCAGCAGGAGG<br>CCCGACGTGTGCCCTCACGACGGCGCCTGCCAACCCTACGAGAAGTG<br>CTGCTTCGACACCTGCCTGAACCACCACACCTGCAAGCTGGCCGACG<br>AGCCCTCCAGCACCGCGACGCCCACGCTCACCACGCCCACCACGAAG<br>CCTCCGTACGTCATCGCCAACTTCGCGAGAAACAAGGTGCATCGAGG<br>AGGA*TAA*	(+)	381	MRVIVLALLVAASSACR<br>YYCKTLSDETYCCDGG<br>KDYNPPEYHDGNCPKV<br>RSFCPRIEGGSRRPDVCP<br>HDGACQPYEKCCFDTC<br>LNHHTCKLADEPSSTAT<br>PTLTTPTTKPPYVIANFA<br>RNKVHRGG	126
DN8354	**ATG**CCCAAGACACACGTCGAAGCAGCACTTGTCAGCAGCCGACAGG<br>CACTGGCCGTCGTGGGCGCAGGCGTTGGGCTGACTGCCTCCGAGGTG<br>GGTCGGTGGACAGCTGGCTCGCACAGCGGGACAGGAGCCGGATCTG<br>ACGACTGGGTAATTTCCTTTACCCTGAGAGACGTCTCGGCCGCAGCA<br>GTACCTCTTGTTGTTCGGTCCCCGGCACCAGACCTTGCACACTCCTTG<br>GCCTCCCTGACTGGGGTAGTGGCCTCCCTGACTGGGATAGTGGCCTC<br>CCTGACTGGGGTAGTGGCCTCCCTGACTGGGATAGTGGCCTCCCTGA<br>CTGGGGTAGTGGCCTCCCTGACT	(-)	351	MPKTHVEAALVSSRQA<br>LAVVGAGVGLTASEVG<br>RWTAGSHSGTGAGSDD<br>WVISFTLRDVSAAAVPL<br>VVRSPAPDLAHSLASLT<br>GVVASLTGIVASLTGVV<br>ASLTGIVASLTGVVASL<br>T	117

DN34505	**ATG**ATGATCCGCCTGCTCCTCCTCGTCACCGTGGCTGCCGTCGTCGTC<br>GCGGCGCAGGGCGGCGGCGACGCCGATGGTTGCAGGTATTACTGCA<br>GGAAGTGGCGGCCGGAGGGAGAAGAGCGGCCCGTGTACTGCTGTGA<br>CGACGGGACGGTTAGCGACCCGCCTCCCGAAGCCGAACACAGTGGA<br>GAATGCCCCGACATTCGGCGTCACTGCCTTCGCAGCAAAAGACCACC<br>AAATGTGTGCCCCCACGACGGTTTCTGCCCGTCCAACCAGAAGTGCT<br>GCTGGGACACCTGCTTGGACCATCATGCTTGTAAACTCCCTGTT*TAA*	(-)	327	MMIRLLLLVTVAAVVV<br>AAQGGGDADGCRYYC<br>RKWRPEGEERPVYCCD<br>DGTVSDPPPEAEHSGEC<br>PDIRRHCLRSKRPPNVC<br>PHDGFCPSNQKCCWDT<br>CLDHHACKLPV	108
DN1553	**ATG**CTGAAGTTTGTAGTATTAGCCGTTGTCGCCGTGGCCGTGGCGCA<br>CGCGCAGGATAAAGACAAGGCCGGCACTCGCTTAGGAGGAGGATTC<br>GGGGTTCCTGGAGCCGGTGGCGTCTTCCCAGGAGCCGGTGGCGTCCC<br>TGGAGTAGGTGGCGTCTTCCCAGGAGCCGGTGGCGTCTTCCCTGGAG<br>CCGGTGGTATCGGTCCTGGACCCGGCGGCCTCATCCCCGGAGGCGGA<br>TTCAACTGCAATTACTGCAGGACGCCCGTCGGGTACGTCTGCTGCAA<br>GCCCGGTAGGTGCCCTCCGGTTCGAGACGTCTGCCCGTCGACCCGCT<br>TCGGACCCCCGGTCTGCCGCCAGGACCTGGACTGCTCCGGCTCCGAC<br>AAGTGCTGCTATGACGTCTGCCTGGAAGACACAGTCTGCAAACCCAT<br>CGTGGCAGGTTCTCAGGGA*TAA*	(-)	444	MLKFVVLAVVAVAVA<br>HAQDKDKAGTRLGGGF<br>GVPGAGGVFPGAGGVP<br>GVGGVFPGAGGVFPGA<br>GGIGPGPGGLIPGGGFN<br>CNYCRTPVGYVCCKPG<br>RCPPVRDVCPSTRFGPP<br>VCRQDLDCSGSDKCCY<br>DVCLEDTVCKPIVAGSQ<br>G	147
DN10332	**ATG**GCTTTGAGTTCAACTTTCCTGAGTGCTGTGTTGGTTGTGGCACTG<br>GTGGCACCGTTAGCGCCGCCGTGCCACGGGTTTTCGCTAAAAGACCT<br>TTTTGTACCTGTTATTAAGGACCAAGTTTCTGATCTATGGAGAACCGG<br>TGACATTGACCTCGTTGGCCACTCCTGCACCTACAATGTCAAGCCGG<br>ATATTGACGGCTTTGAACTCTACTTCATCGGGTCCGTCACGTGTCCGG<br>GCTGGACTACGCTCAGGGGAGAGTCCAACACCCGCAGCAAATCCGG<br>TGTTGTGAACGCCGCTGTGAAAGACTTCATTCAGAAAGCTCTCAAAG<br>CAGGTCTGGTCACCGAAGAAGAAGCCAAACCACACCTCGTA*TAA*	(-)	375	MALSSTFLSAVLVVALV<br>APLAPPCHGFSLKDLFV<br>PVIKDQVSDLWRTGDID<br>LVGHSCTYNVKPDIDGF<br>ELYFIGSVTCPGWTTLR<br>GESNTRSKSGVVNAAV<br>KDFIQKALKAGLVTEEE<br>AKPHLV	124

DN2676	**ATG**TCAAGTATCCCTCAAGGCTTTCTGACCTGCGTGGTGAAGGCTTC<br>CTACAAGAGAAGTATATACAATTCGAGTGACGTCATGACGAATCTGC<br>GAACTCCATGGACTCACTGGCTGACACTGCTACTGCTGATGGCGACC<br>AGCATGATGCTACTGTCAGCCCAGGAGATGGAAGACCAAGAAAATT<br>ACGCTTCCGATATTTTCTCCAAAATTTTTAATTCCCTGGTCAAAGATG<br>GCGAGATCGAGCTTCTGGGTCACTACTGTTCTTATTCCACGCGCCCTT<br>ACTTTCTTCGATGGCGGCTCAAGTTCAAGAGCAAAGTCTGGTGCCCG<br>GGTTGGACGCTCGTCTACGGCAGCGCCAGTGAAAGCTCCAGTGTGTC<br>CAACAGCATTCAAAACGCCATCATCAACTTCATCCAGAAAGCTTACC<br>AAGAAGGTGTCATAACCGAGGAGGATGCAAAGCCATGGTTGCAGGG<br>GAGCCAT*TGA*	(+)	480	MSSIPQGFLTCVVKASY<br>KRSIYNSSDVMTNLRTP<br>WTHWLTLLLLMATSM<br>MLLSAQEMEDQENYAS<br>DIFSKIFNSLVKDGEIEL<br>LGHYCSYSTRPYFLRW<br>RLKFKSKVWCPGWTLV<br>YGSASESSSVSNSIQNAI<br>INFIQKAYQEGVITEEDA<br>KPWLQGSH	159
DN13227	**ATG**GTTATCACTAGAGACTCGGCAATTCTAACTGCTGAACTGAACTC<br>ATCGGGACACTGTGGCAGCCGGAACAGCGTCAAACAGACAGTCAGC<br>GTTAAGAGAGAGGTTTCCCCTCCTTCAGGAACTTCGATTCATAACAC<br>TTTCAAGATGCGAGTGTCTGTCCTCAGCATGGCCCTCGTGGTGGCGTT<br>GGCTGCGTCCCTCGCGCCGCAGTGCCAAGCGAGTGGCTGGGAGGCG<br>CTGGTGCCGGCCATTGCGAACAAACTCACTGGACTGTGGGAGAGCG<br>GAGAGCTGGAGCTGTTAGGACACTACTGCAACTTTAGCGTGACACCG<br>AAATTCAAGCGCTGGCAACTGTATTTCAGGGGTCGCATGTGGTGCCC<br>AGGATGGACAGCCATCAGAGGCCAAGCCGAGACCCGTAGCAGATCG<br>GGCGTGGTAGGCAGAACGACGCAGGACTTCGTCAGGAAAGCTTTCA<br>GCGCGGGTCTCATCACCGAATCAGAAGCCCAAGTTTGGCTTAATAGT<br>*TAA*	(-)	516	MVITRDSAILTAELNSS<br>GHCGSRNSVKQTVSVK<br>REVSPPSGTSIHNTFKM<br>RVSVLSMALVVALAAS<br>LAPQCQASGWEALVPAI<br>ANKLTGLWESGELELL<br>GHYCNFSVTPKFKRWQ<br>LYFRGRMWCPGWTAIR<br>GQAETRSRSGVVGRTT<br>QDFVRKAFSAGLITESE<br>AQVWLNS	171
DN4554	**ATG**AAACTCGTTGTGTTCATATCACTGGTCGGTTTGGTCCTAACCGA<br>AAATGTTAATACGAAAGAGAAGTTATTAACCTATATTGCTCAGGAAC<br>TTACGTGGCATGGAAGGAACGGCAGCGTGACATTCCTCCACAACAA<br>ATGCGAATTTTCCGTGACTCCGAAGTCGAAGGACTGGATGCCTTATC<br>ATGAGAGCAATTTCAGTTGCCCCGACTGGACAAACATTGTAGGAGAA<br>GCCGAGGGACGCTGTCGGGTGATCACTGCAGCGAAAGCCGCTAAGG<br>ATTTCGTTGTTCGGGCTCTGGATATTGGCCTCTTCAACTTCTATGATG<br>GAAAAGCATGGCTCTTTTCCGAGATAGCAACTGACACAAACAATATC<br>ATGTTATCCTTA*TAA*	(+)	390	MKLVVFISLVGLVLTEN<br>VNTKEKLLTYIAQELTW<br>HGRNGSVTFLHNKCEFS<br>VTPKSKDWMPYHESNF<br>SCPDWTNIVGEAEGRCR<br>VITAAKAAKDFVVRAL<br>DIGLFNFYDGKAWLFSE<br>IATDTNNIMLSL	129

DN19134	**ATG**GCGCCAACTACATCCAGTAGAGTTGCCAAGTTTTCCATTATCTG<br>TTTCCTGTTATTCGCCTCTGTCTCAGCGCGCCCTCAGCTCGGAGACGT<br>CCTTGGATCTGTCGTTGAAACTTTCCTGAAGAATGCAGTCAAACCTC<br>AGAACTCACGATTCTTGATAACTATTGCTACG*TGA*	(+)	177	MAPTTSSRVAKFSIICFL<br>LFASVSARPQLGDVLGS<br>VVETFLKNAVKPQNSRF<br>LITIAT	58
DN468	**ATG**CGTGTCTCCGTGTTGACAAGCCTGGTGGTGGCGGTGTTCCTGGT<br>GGCACTCTTCGCCCCAGAGTGCCAGGCGCAAGGATGGCAGGCTGTG<br>GCAGCGGCCGTCGCCAGCAAGATCGTTGGGCTGTGGAGGAACGAGG<br>AGACGGAGCTGCTGGGACATAAGTGCCGCTTCACCGTCAAACCTTAC<br>ATCAAGAGGTTACAGCTGAACTACAAGGGGAAGATGTGGTGCCCCG<br>GCTGGACGACTATCAGAGGGGAAGCCAGGACACGCAGCCATTCCGG<br>GGTGGCTGGAAGGACGGCCAGGGACTTCGTCGAGAAAGCCTTCAGG<br>GACGGCCTCATCTCCGAACAAGATGCTAAGCGGTGGCTGAAC*TAA*	(+)	369	MRVSVLTSLVVAVFLV<br>ALFAPECQAQGWQAVA<br>AAVASKIVGLWRNEET<br>ELLGHKCRFTVKPYIKR<br>LQLNYKGKMWCPGWT<br>TIRGEARTRSHSGVAGR<br>TARDFVEKAFRDGLISE<br>QDAKRWLN	122
DN63324	**ATG**GCTTTATCGACGAAGTCTCGCATAGATTCCTCTGCCGTCGCTAG<br>TTTGCAGCGCGACGAGGCTTTGCCTGTGATATTAGGCCAATGGTCAC<br>AGGTGAAAGTGCACTGGTGGTACACAGTCCAGTGGGAGAACCTATG<br>CTCCACTTCAATCTTGCATGAGTGTCCCATGAAAGTCACGCTTTTGTT<br>CATCCAGTGCCCAAGAAGATGCCGAGCAACCTCTTTTGTAAGTTTCC<br>CCTTTTTCTTTGCGTTGCAGTGCGCGGCCAAGACCAGGACAAGGAGA<br>GTGATCATCACAGACGGTCTCATGATGGTTATCTACACAGAA*TAG*	(-)	327	MALSTKSRIDSSAVASL<br>QRDEALPVILGQWSQV<br>KVHWWYTVQWENLCS<br>TSILHECPMKVTLLFIQC<br>PRRCRATSFVSFPFFFAL<br>QCAAKTRTRRVIITDGL<br>MMVIYTE	108

Start codons are indicated as bold texts while stop codons were written in italic font if detected, the identified crptide in each precursor was indicated as underlined text.

Table S.2 Functional annotation of the *in silico-identified* precursors. The detected amino acid sequences for the selected gene-encoded AMPs were subjected as query sequences to the online BLASTp tool.

Trinity ID	BLASTp top hit	Accession No.	Query coverage	Identity	E. value
DN1899	Uncharacterized protein (*P. vannamei*)	XP_027219394.1	100 %	94 %	3E-106
DN35987	Uncharacterized protein (*P. vannamei*)	XP_027212782.1	100 %	98%	4E-86
DN8354	hypothetical protein (*Fictibacillus enclensis*)	WP_061975429.1	33%	76%	2.00E-04
DN34505	Pl-crustin 2 [*Penaeus vannamei*]	ROT80205.1	100%	98.15 %	5e-72
DN1553	Crustin-like protein (*P. vannamei*)	ROT68344.1	100 %	100%	2e-95
DN10332	anti-lipopolysaccharide factor 3 precursor (*Penaeus vannamei*)	QJD13445.1	100%	100%	2e-85
DN2676	anti-lipopolysaccharide factor-like (*P. vannamei*)	XP_027219859.1	100 %	99.3 %	4E-114
DN13227	anti-lipopolysaccharide factor-like(*P. vannamei*)	XP_027217591.1	71 %	100 %	5E-84
DN4554	anti-lipopolysaccharide factor-like (*P. vannamei*)	XP_027214209.1	100 %	99.2 %	7E-91
DN19134	anti-lipopolysaccharide factor-like (*P. vannamei*)	XP_027239649.1	100 %	100 %	4E-87
DN468	anti-lipopolysaccharide factor-like (*P. vannamei*)	XP_027206930.1	100 %	100 %	1E-84
DN63324	Anti-lipopolysaccharide factor isoform 2 (*P. vannamei*)	ROT72330.1	100 %	64.8 %	5E-34

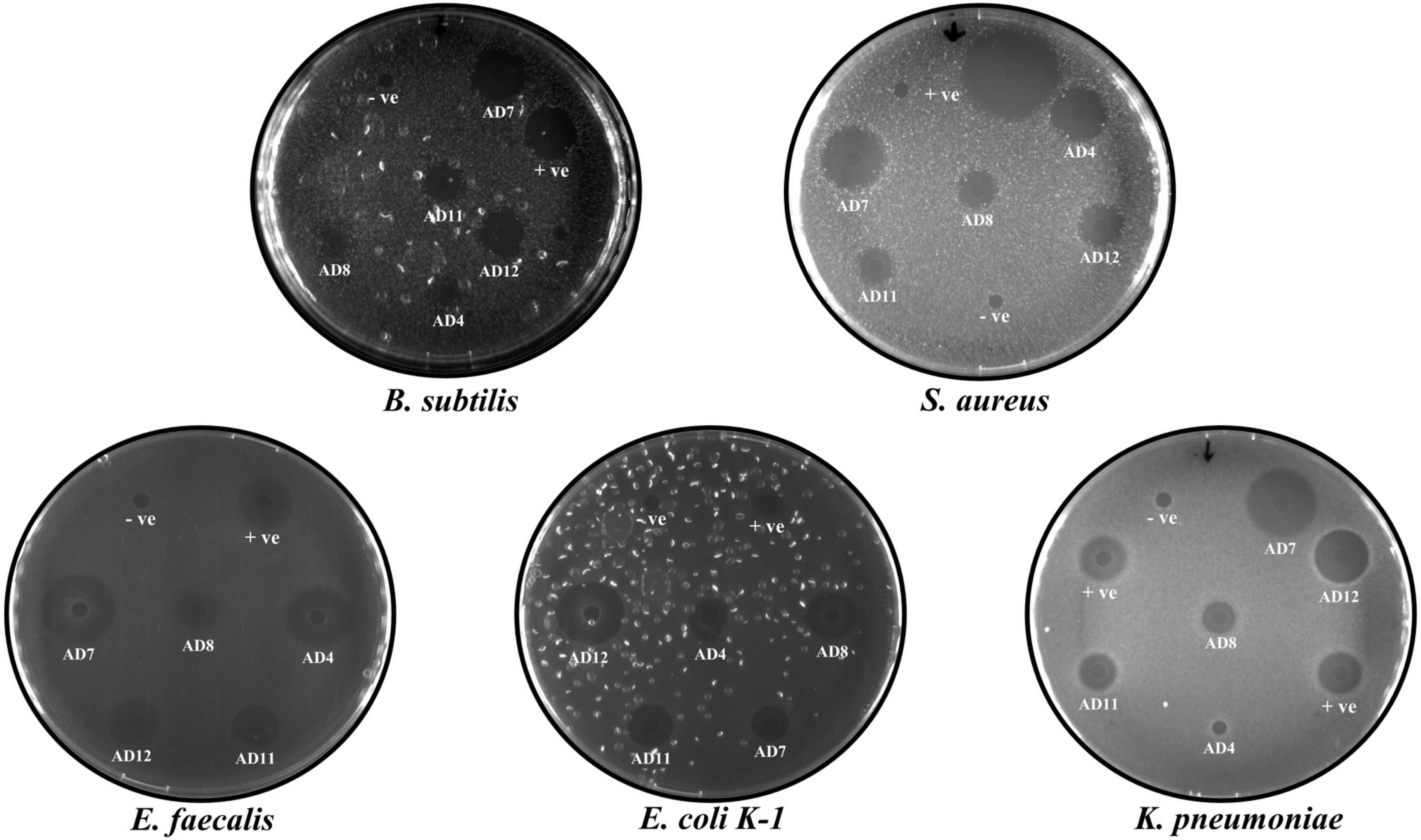
- ve
AD7
+ ve
AD11
AD8
AD12
AD4
B. subtilis
+ ve
AD4
AD7
AD8
AD12
AD11
- ve
S. aureus
- ve
+ ve
AD7
AD8
AD4
AD12
AD11
E. faecalis
- ve
+ ve
AD12
AD4
AD8
AD11
AD7
E. coli K-1
- ve
AD7
+ ve
AD12
AD8
AD11
+ ve
AD4
K. pneumoniae

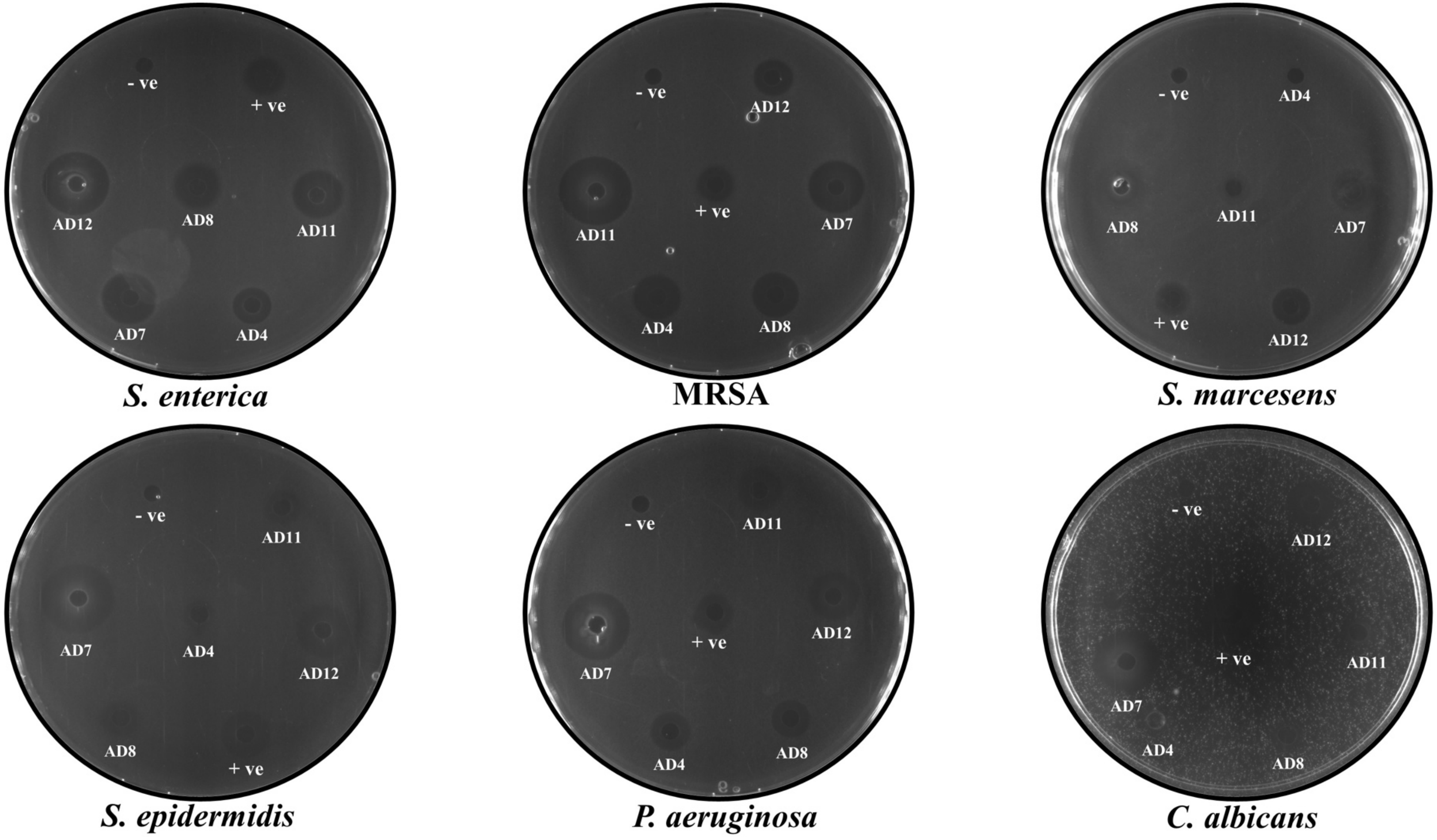

Figure S.2 Radial Diffusion Assay. Each indicator pathogen was inoculated into 10 ml of the underlay agarose gel at the density of 4×10^6 CFU, during the mid-logarithmic phase, and poured into a 100 mm petri dishe. Wells with 3 mm diameter were punched and 5 µl of 500 µM stock solution from each peptide was added to each well. Plates were incubated at 37 °C for 3 h for the diffusion of peptides then the underlay gel was covered with 10 mL of nutrient-rich overlay and the plates were then incubated for 12h at 37 °C. The antimicrobial activities were measured by the absolute unit (A.U).

Table S.3 Antimicrobial activity spectra of the selected cryptides. Five microliters of 500 µM of each cryptide were dispensed into 3 mm-diameter wells, that were punched in the pre-inoculated underlay agarose layer. After diffusion, 10 ml of a nutrient-rich layer was added to each plate. The plates were then incubated for 12 h at 37 °C and antimicrobial activities were measured in the term of absolute unit (A.U).

Indicator pathogens	**Peptide ID**						
	**AD4**	**AD7**	**AD8**	**AD11**	**AD12**	**Melittin**	0.01% Acetic acid
B. subtilis	13.8± 0.7	22.8 ± 0.9	18.3 ± 0.8	21.7 ± 1.5	22.8 ± 0.9	21.8± 3.1	0
S. aureus	24.4 ± 0.9	31.4 ± 1	12.6 ± 1.3	10.4 ± 1.2	18.3 ± 0.8	41.5 ± 2.5	0
E. faecalis	14.7 ± 1.2	17.3 ± 1.3	6.2 ± 0.8	12.2 ± 1.1	13.4 ± 0	11.8 ± 1.3	0
E. coli K-1	7.7 ± 1	8.3 ± 1	9 ± 0	8.3 ±1	11.8 ± 1.3	6.5 ± 0.9	0
K. pneumoniae	0	20.2 ± 1.5	9 ± 0	11.1 ± 2.1	16.4 ± 0.8	11.8 ± 1.3	0
S. enterica	7.7 ± 1	10.4 ± 1.2	9 ± 0	9.3 ± 0.6	11.8 ± 1.3	7.8 ± 2	0
MRSA	12.2 ± 1.1	14.7 ± 1.2	11.1 ± 2.1	14.7 ± 1.2	10 ± 1	11.8 ± 1.3	0
S. marcescens	0	6.2 ± 0.8	4 ± 0	4 ± 0	7.7 ± 1	7.7 ± 1	0
S. epidermidis	9 ± 0	16 ± 0	10 ± 1	10 ± 1	14.7 ± 1.2	10.4 ± 1.2	0
P. aeruginosa	0	9 ± 0	2.7 ± 5.4	6.2 ± 0.8	8 ± 0.9	6 ± 0.9	0
C. albicans	2.1 ± 0.5	17.8 ± 1.6	3.1 ± 0.7	2.4 ± 0.5	6.5 ± 0.9	14.2 ± 1.4	0

Antibacterial Activity (AU) ± SD

Table S.4 Antibiofilm activities of the selected Cryptides against *S. enterica* and MRSA. Microbial cells were suspended at 1.7 X 108 CFU/ml density, during the mid-logarithmic phase in MHB. Then, 96 U-shaped -well plates were inoculated with 100 µl of microbial suspension and incubated for 48 h at 37 °C. The formed biofilms were washed with 10 mM Tris-HCl and treated with 100 µl of different concentrations of each peptide for 24 h. The treated biomasses were washed and fixed with methanol for 15 min at -20 °C before staining with 0.1 % crystal violet. The stained biofilms were dissolved in 100 µl of 33 % acetic acid and the quantities of crystal violet stains were determined by measuring absorbance at 600 nm as an indicator of the remaining biomass after treatment. Untreated cells were used as negative control [indicating 100 % biofilm integrity] while the sterile medium was used as a positive control [indicating 0 % biofilm integrity].

Conc. (µM)	CRYPTIDES									
	AD4		AD7		AD8		AD11		AD12	
	S. enterica	MRSA	*S. enterica*	MRSA	*S. enterica*	MRSA	*S. enterica*	MRSA	*S. enterica*	MRSA
**12.5**	6.16 ± 2.7	0	23.79 ± 3.4	2.46 ±2	8.86 ± 1.9	11.78 ± 4.3	7.07 ± 1.1	12.91 ± 3.6	5.78 ± 2.2	3.74 ± 2.9
**25**	12.58 ± 1.9	0.86 ± 3.1	54.63 ± 1.7	47.51	30.93 ± 2.2	29.35 ± 1.2	22.89 ± 2.6	16.26 ± 1.1	32.23 ± 0.9	25.68 ± 2.9
**50**	30.41 ± 1.9	11.53 ±1.4	65.63 ± 1.8	62.03 ± 0.6	58.31 ± 3.6	46.17 ± 3.2	50.28 ± 2.8	33.79 ± 0.8	60.45 ± 1.9	35.24 ± 3.6
**100**	45.13 ± 2	27.05 ±1.3	100	69.07 ± 1.2	96.01± 3.4	66.98 ± 1.3	80.08 ± 3.4	41.78 ± 0.7	83.39± 0.6	51.96 ± 4
**200**	80.63 ± 1	35.86 ± 2.5	100	77.97 ± 2.4	100	83.07 ± 0.6	100	62.12 ± 0.8	100	72.84 ± 0.6

Biofilm eradication % ± SD

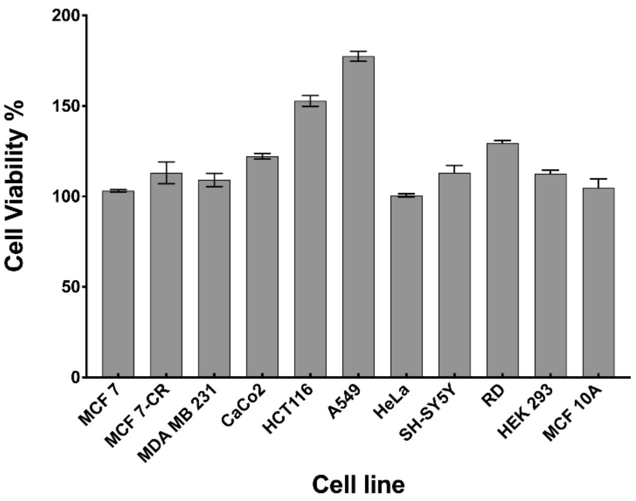

Figure S.3 The effect of serum deprivation on the selected cell lines. Cells were seeded and maintained until reached 75-80 % confluency into complete media. Then, the cells were incubated in serum-free media for 24 h under the same conditions. Cell viability percentages in the absence of serum were measured by MTT assay. All experiments were done in triplicates and the averages were calculated.
